# Feasible-metabolic-pathway-exploration technique using chemical latent space

**DOI:** 10.1093/bioinformatics/btaa809

**Published:** 2020-12-29

**Authors:** Taiki Fuji, Shiori Nakazawa, Kiyoto Ito

**Affiliations:** Center for Exploratory Research, Research and Development Group, Hitachi, Ltd., Kokubunji-shi, Tokyo 185-8601, Japan

## Abstract

**Motivation:**

Exploring metabolic pathways is one of the key techniques for developing highly productive microbes for the bioproduction of chemical compounds. To explore feasible pathways, not only examining a combination of well-known enzymatic reactions but also finding potential enzymatic reactions that can catalyze the desired structural changes are necessary. To achieve this, most conventional techniques use manually predefined-reaction rules, however, they cannot sufficiently find potential reactions because the conventional rules cannot comprehensively express structural changes before and after enzymatic reactions. Evaluating the feasibility of the explored pathways is another challenge because there is no way to validate the reaction possibility of unknown enzymatic reactions by these rules. Therefore, a technique for comprehensively capturing the structural changes in enzymatic reactions and a technique for evaluating the pathway feasibility are still necessary to explore feasible metabolic pathways.

**Results:**

We developed a feasible-pathway-exploration technique using chemical latent space obtained from a deep generative model for compound structures. With this technique, an enzymatic reaction is regarded as a difference vector between the main substrate and the main product in chemical latent space acquired from the generative model. Features of the enzymatic reaction are embedded into the fixed-dimensional vector, and it is possible to express structural changes of enzymatic reactions comprehensively. The technique also involves differential-evolution-based reaction selection to design feasible candidate pathways and pathway scoring using neural-network-based reaction-possibility prediction. The proposed technique was applied to the non-registered pathways relevant to the production of 2-butanone, and successfully explored feasible pathways that include such reactions.

## 1 Introduction

Microbial production of chemical compounds is an important contributor to promote sustainable industries. Since the development of a highly productive microbe often requires a huge amount of time and effort, technologies for designing and constructing biological functions of microbes on computers have become increasingly important to shorten the development period of such microbes. An essential step in *in silico* microbial design technologies is metabolic-pathway design in which a series of enzymatic reactions that promote desired chemical structural changes from a start compound (metabolite) to a target compound are determined (Choi *et al.*, 2019). In addition to the selection of intermediate compounds, potential enzymes catalyzing chemical reactions among the intermediate compounds should be found. Namely, to design highly productive metabolic pathways, not only examining a combination of well-known enzymatic reactions but also finding a combination of potential enzymatic reactions that can catalyze the desired structural changes are necessary. Since it takes much manual and computational effort to explore all feasible metabolic pathways that include such potential reactions, an efficient technique for exploring such pathways is still necessary to shorten the development time of highly productive microbes. 

Although various *in silico* metabolic-pathway-exploration techniques have been proposed (Wang *et al.*, 2017), three major technical challenges for efficient metabolic-pathway exploration remain; (i) how to represent an enzymatic reaction on a computer system, (ii) how to design feasible-candidate pathways by combining a huge number of potential enzymatic reactions and (iii) how to evaluate the relevance of the feasible-candidate pathways. For the first challenge, most conventional techniques use a reaction−representation method that involves manually preparing reaction rules defined in advance and determining changes in the substructure focusing near the reaction center based on the reaction rules (Araki *et al.*, 2014; Delépine *et al.*, 2018; Hadadi and Hatzimanikatis, 2015; Kumar *et al.*, 2018; Moriya *et al.*, 2010). While this representation method accurately identifies small changes in partial structures such as a functional group, they do not sufficiently identify the overall backbone structures involved in the substrate specificities of enzymatic reactions. For the second challenge, conventional techniques often explore feasible pathways by using rule-based logical operations such as adding and removing functional groups or atoms. They have an advantage in that unrealistic pathways that include unrealistic compound structures and enzymatic reactions are not explored. However, feasible pathways are not sufficiently explored because these techniques do not take into account enzymatic reactions not existing in the operation rule. For the third challenge, validation of the relevance of unknown enzymatic reactions also becomes a problem with conventional rule-based techniques. Therefore, chemical embedding that can quantify the feature of compound structures more precisely than conventional techniques, such as variational autoencoder (VAE), is necessary.

We propose a feasible-metabolic-pathway-exploration technique using the chemical latent space acquired from a deep generative model for compound structures. The deep generative model for compounds was recently proposed to map a compound structure described in simplified molecular input line entry system (SMILES) styles to a latent vector space (Gómez-Bombarelli *et al.*, 2018; Jin *et al.*, 2018; Kusner *et al.*, 2017). By using the chemical latent space, this technique involves a method with which enzymatic reactions are represented as a difference vector between the latent vectors of a main substrate and that of a main product. By using metabolic reaction representation, it is possible not only to determine changes in the overall backbone structure related to substrate specificity but also to eliminate the need for reaction rules that have been required for each reaction so that reactions can be performed uniformly. Thanks to the identical dimensions of the latent vectors, the latent vector(s) of intermediate compound(s) between the start and target compounds can be expressed by simple mathematical operation among the reaction-feature vectors. Moreover, the latent vector of each intermediate compound can be reconstructed using the deep generative model and used for new compound structures. We also developed a differential evolution (DE)-based feasible-pathway-design technique of candidate pathways by combining potential enzymatic reactions, and a neural network (NN)-based-reaction-possibility prediction method to evaluate the relevance of potential enzymatic reactions and feasible pathways as candidate pathways. This design technique selects the reaction-feature vector(s) and minimizes the squared error between the pathway-feature vector, which was calculated by the latent vectors of the start and target compounds, and the sum of selected reaction-feature vectors. The scoring method calculates the reaction-possibility value and overall pathway score of each reaction in consideration of the substrate specificity in the latent space. To verify the effectiveness of the proposed technique, we applied it to pathway-exploration problems that include both registered and non-registered reactions.

## 2 Materials and methods

### 2.1 Feasible-metabolic-pathway exploration and related work

**Fig. 1.**
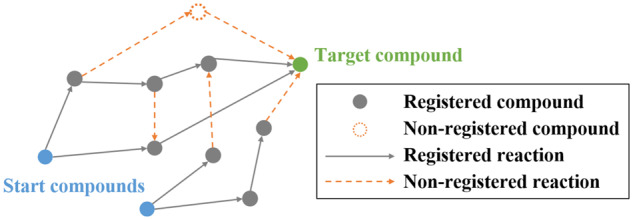
Feasible-metabolic-pathway exploration. There are often more than one pathway for producing target compound from start compound in metabolic system. In addition to such known pathways, unknown enzyme reactions and compounds that are not registered in database (DB) may be included. Namely, there may be several feasible pathways from start compound to target compound that include both registered (gray solid lines) and non-registered (orange dotted lines) reactions

Exploration of metabolic pathways involves discovering a series of enzymatic reactions that promote a desired structural change in chemicals from a start compound to a target compound. During the exploration, it is necessary not only to explore all the pathways consisting of several known enzymatic reactions registered in curated biological pathway databases (DBs), such as Kyoto Encyclopedia of Genes and Genomes (KEGG) (Kanehisa and Goto, 2000), Metacyc (Caspi *et al.*, 2018) and MetaNetX (Moretti *et al.*, 2016) but also feasible pathways composed of enzymatic reactions that have not been registered in the DBs but potentially catalyze intermediate compounds (Fig. 1). 

For efficient exploration of such feasible pathways on computer systems, it is useful to use a method with which each enzymatic reaction is regarded as an arithmetic expression, i.e. vectors or matrices, and an entire pathway can be computed as an arithmetical superposition of individual reactions. By using such a mathematical method, a structural change between a substrate and product catalyzed by an enzymatic reaction can be expressed as an addition, substitution or rearrangement; thus, a variety of potential enzymatic reactions and intermediate compounds can be easily created and explored using computational algorithms.

Reaction representations, such as molecular fingerprints, based on certain substructure-counting methods, that represent a compound structure with a vector consisting of the number of defined partial structures constituting the whole structure have been proposed (Araki *et al.*, 2014; Kumar *et al.*, 2018). They also define a difference vector obtained by subtracting the structural-feature vector of the main substrate from a main product as a reaction feature and associated with the Enzyme Commission (EC) number by using the metabolic pathway information of KEGG. Therefore, it is possible to acquire the structural-feature vectors of a product compound by adding an arbitrary reaction-feature vector to the structural-feature vectors of the substrate compound.

One problem in such a conventional fingerprint-based vector representation is that a molecular fingerprint vector cannot reproduce a compound structure because it does not have information on the connectivity among partial structures. In the case of a fingerprint of an unknown compound that is catalyzed by potential enzymes, it is only possible to specify a known similar compound structure by performing a structure search. Furthermore, in the case of compounds having different absolute configurations, i.e. isomers, even known compounds cannot be distinguished. To solve this problem, it is necessary to develop a pathway-exploration technique using another mathematical method of enzymatic reactions that satisfies the following two points:

- Compounds in the metabolic pathway can be expressed in a distributed representation with a feature space of a fixed dimension.
- A structure-feature vector of a product after adding a reaction-feature vector to a structure-feature vector of a substrate can be decoded to a compound structure without losing information on the connectivity.

A deep generative model for chemical compounds, known as a molecular autoencoder, is an innovative technique of compound expression based on the variational Bayesian method, in which strings of the SMILES of compounds are encoded to a fixed dimension of latent vectors (Gómez-Bombarelli *et al.*, 2018). To satisfy the above requirements, therefore, the proposed technique uses latent vectors based on the junction-tree VAE (JT-VAE), which is a state-of-the-art deep generative model for chemical compounds (Jin *et al.*, 2018).

### 2.2 Proposed feasible-pathway-exploration technique

#### 2.2.1 Overall structure

**Fig. 2.**
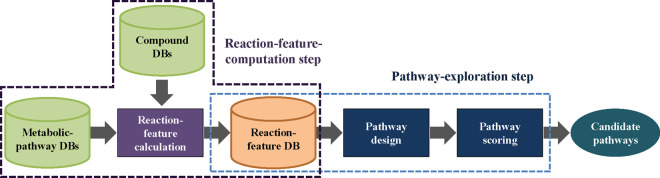
Overview of proposed technique. It involves reaction-feature-computation and pathway-exploration steps. In reaction-feature computation, variational autoencoder (VAE) models are trained with public compound DB. By using latent vectors of compounds, reaction-feature vectors are then calculated. Pathway exploration consists of pathway design and pathway scoring. Namely, several candidate pathways are designed and scored

Figure 2 illustrates the overall structure of the proposed technique. The technique is roughly divided into two steps: reaction-feature computation and pathway exploration. In the first step, reaction features of compounds on a metabolic pathway are computed as feature vectors by using a deep generative model and accumulated in the reaction-feature DB. Pathway exploration consists of reaction-feature selection in which candidate pathways are explored using the feature vectors stored in the reaction-feature DB and pathway scoring in which the most relevant pathway is selected from the candidate pathways. 

#### 2.2.2 Reaction-feature vectors using chemical latent space

**Fig. 3.**
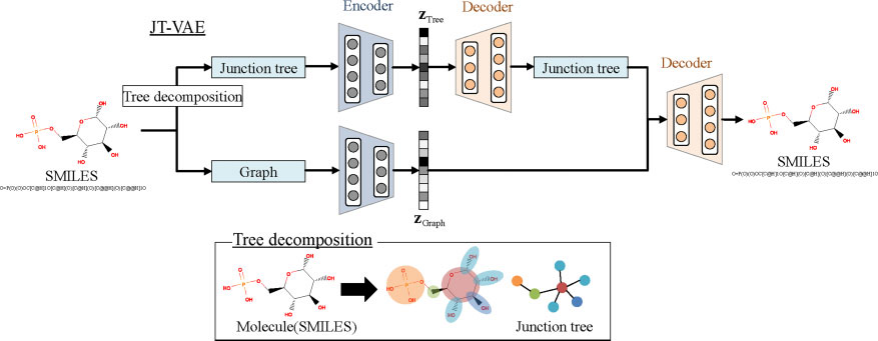
Architecture of junction tree VAE (JT-VAE) (Jin *et al.*, 2018). JT-VAE has two encoders, graph and tree. Input of tree encoder is junction tree decomposed using feature-tree technique (Rarey and Dixon, 1998). Color node in feature tree represents substructure of compound

As mentioned above, we use encoders based on the JT-VAE to encode a certain structure of a chemical compound into a latent vector. Figure 3 shows an overview of the JT-VAE. It has two types of encoders. One is a graph encoder and the other is a tree encoder. Tree decomposition on the basis of the feature-tree technique (Rarey and Dixon, 1998) is carried out for evaluating the molecular similarity between small organic compounds. Instead of a linear representation, such as fingerprints, a more complex description, a feature tree, is calculated for a molecule. Such a characteristic of the junction tree is effective for representing the overall backbone structure of compounds; thus, the tree-latent vector encoded with the JT-VAE is expected to also represent the compound structure. 

**Fig. 4.**
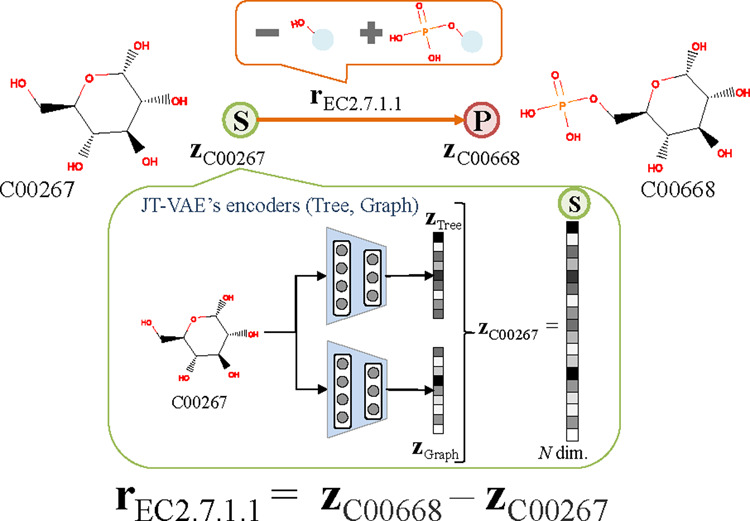
Explanation of reaction-feature vector. First, latent vectors of compounds registered in metabolic-pathway DBs are acquired from JT-VAE encoders. Then, by using latent vectors of main substrate and product on basis of metabolic-pathway DBs, reaction-feature vector, which is defined as difference vector of these latent vectors, is obtained. Reaction-feature vector of EC2.7.1.1 subtracts hydroxy group and adds phosphate group to *α*-d-glucose

Figure 4 illustrates the method for generating reaction-feature vectors by using the encoders of the JT-VAE. First, the encoders are trained using a compound dataset in a compound DB before computing the reaction-feature vectors. Then, metabolic-pathway information, such as ‘glycolysis’, is parsed from a metabolic pathway DB, such as KEGG. Next, an SMILES string of a compound on the metabolic pathway is input to the trained encoders of the JT-VAE. A latent vector ($z_{C00267}$) of the compound is generated and mapped to a latent space of *N*-dimensions. A reaction-feature vector is generated by subtracting the latent vector of the main substrate from that of the main product with the following equation:

(1) $$r_{\mathrm{ec}}=z_{\mathrm{pro}}-z_{\mathrm{sub}}$$

where $z_{\mathrm{pro}}$ is a latent vector of a product compound and $z_{\mathrm{sub}}$ is that of a substrate compound, as shown in Figure 4. 

In this manner, all reactions on metabolic pathways in the metabolic-pathway DB are encoded to reaction-feature vectors and stored in the reaction-feature DB. Simultaneously, each reaction-feature vector is recorded and assigned an EC number. In Figure 4, e.g. the reaction-feature vector $r_{\mathrm{ec}2.7.1.1}$ generated from the latent vectors $z_{C00267}$ and $z_{C00668}$ of the main substrate *α*-d-glucose (KEGG Compound ID: C00267) and main product *α*-d-glucose 6-phosphate (KEGG Compound ID: C00668) is recorded with the EC number of 2.7.1.1.

#### 2.2.3 Pathway design of candidate pathways

**Fig. 5.**
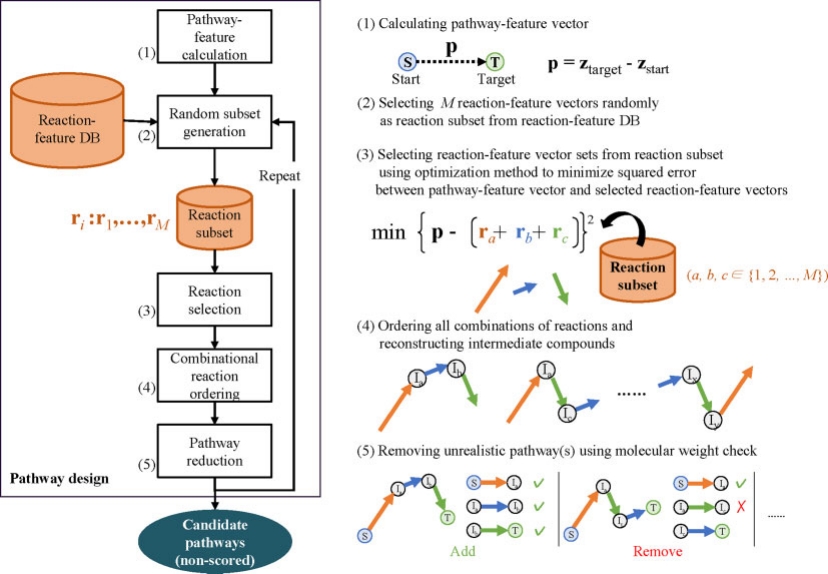
Procedure of pathway design of candidate pathways. Reaction-feature-vector sets are selected using optimization method to minimize squared error between pathway-feature vector and sum of selected reaction-feature vectors. This figure illustrates example in which three reaction-feature vectors ($r_{a},\;r_{b}$ and $r_{c}$) are selected. There are total of six combinational orders. Intermediate compounds are reconstructed using JT-VAE decoder. Finally, unrealistic pathway(s) is removed based on molecular weight changes, and remaining candidate pathways are added to candidate-pathway list

Figure 5 shows the procedure of the pathway-design step of candidate pathways. This step consists (i) pathway-feature calculation, (ii) random-subset generation, (iii) reaction selection, (iv) combinational-reaction ordering and (v) unrealistic-pathway removal. 

First, a pathway-feature vector **p** is computed as a difference vector obtained by subtracting the latent vector of a target compound $z_{\mathrm{target}}$ from a start compound $z_{\mathrm{start}}$ (Fig. 5(1)). Namely, the pathway-feature vector **p** is derived from the following equation:

(2) $$p=z_{\mathrm{target}}-z_{\mathrm{start}}.$$

Next, *M* reaction-feature vectors are randomly selected from the reaction-feature DB. The selected vectors are defined as a reaction subset to reduce the calculation amount and enhance search efficiency (Fig. 5(2)).

Then, a set of reaction-feature vectors for designing pathways are determined using an optimization method to minimize the squared error between the pathway-feature vector and sum of the reaction-feature vectors in the set (Fig. 5(3)). In other words, the objective function of the optimization is defined with the following equation:

(3) $$\begin{array}{ccc} \min\;|e{|}^{2} & = & \sum_{j=0}^{N-1}{\left(p_{j}-\sum_{i=0}^{M-1}x_{i}r_{i,j}\right)}^{2}\\ & s.t. & |e|\leq \mathrm{Th},\\ & & \sum_{i=1}^{M}x_{i}\leq K,x_{j}\in \{0,1,2,...,K\} \end{array}$$

where *p_j_* is a value for the pathway-feature vector **p** of *j*th dimension, *x_i_* is a integer value for the *i*th subset index, $r_{i,j}$ is a reaction-feature vector’s value of the *i*th subset index and *j*th dimension, Th is the error threshold and *K* is the maximum number of reaction steps.

Since the objective function uses the square error **e** between the sum of the selected reaction-feature vectors and pathway-feature vector, a penalty function that increases non-linearly according to the maximum number of reaction steps is provided. Namely, the minimization problem is dealt with as a non-linear integer programming (NLP) problem.

To solve this NLP problem, we apply a DE technique (Storn and Price, 1997) for its high search performance despite it being a simple algorithm. We introduce a fitness function f(x) into the DE technique, which is derived from the square error $|e{|}^{2}$ among the feature vectors and a penalty function *λ* that increases non-linearly according to the maximum number of reaction steps provided as a constraint condition.

(4) $$\min\;f(x)=|e{|}^{2}+\lambda \to \min$$

 

(5) $$\lambda =\left\{\begin{array}{cc} C\times \;\text{exp}\;(x_{\mathrm{len}}{)}^{2} & \left(x_{\mathrm{len}}>K\right)\\ 0.0 & \left(x_{\mathrm{len}}\leq K\right) \end{array}\right.$$

where *x*_len_ is the number of selected reactions and *C* is a constant parameter. Each individual in the initial population is initialized such that the sum of the elements is within the maximum number of reaction steps. A binary DE algorithm is used in which the value of each element after the evolution calculation is rounded off to handle as an NLP problem. The binary DE algorithm for reaction-feature selection is described as Algorithm 1. By applying the binary DE algorithm to reaction-feature selection, a set of reaction-feature vectors is obtained as a set of component vectors for designing the desired pathway-feature vector.

Algorithm 1 Binary DE algorithm for reaction selection

Initial population P(*g *=* *0) is *P* individuals generated randomly

Evaluate P(*g *=* *0)

Set individual’s length as reaction subset size *M*

**for** generation *g *=* *1 to *Terminate* **do**

 **for** individual *p *=* *0 to *P* – 1  **do**

  Generate random numbers *a*, *b*, *c* ∈ [0, *P* – 1]

  Select three individuals $x_{a},\;x_{b},\;x_{c}$ as parents

  **for** parameter *j *=* *0 to *M* – 1  **do**

   Calculate mutator $v_{j}=x_{a,j}+F(x_{b,j}-x_{c,j})$

   Modify value to binary 0 or 1 as follows:

   **if**  $v_{j}>=0.5$  **then**

    Set $v_{j}=1.0$

   **else**

    Set $v_{j}=0.0$

   **end if**

   Compute crossover as follows:

   Generate uniform random rnd≡U(0,1)

   **if** *rnd* < *CR* **then**

    Set *u_k_* = *v_j_*

   **else**

    Set $u_{k}=x_{p,j}$

   **end if**

  **end for**

  **if**  f(u) <$f(x_{p}$) **then**

   Replace $x_{p}$ with **u**

  **end if**

 **end for**

**end for**

All candidate pathways are then constructed by ordering all combinations of the reaction-feature vectors in the set (Fig. 5(4)). Simultaneously, intermediate compounds in the candidate pathways are reconstructed using the decoder of the JT-VAE. Namely, the reaction-feature vectors are sequentially added to the latent vector of the start compound while obtaining latent vectors of intermediate compounds at each segment in the candidate pathways. These latent vectors of the intermediate compounds are then reproduced as a compound structure SMILES string by the decoder of the JT-VAE.

Finally, we evaluate the candidate pathways and remove unrealistic ones in the following manner (Fig. 5(5)). In the ordering process of the reaction-feature vectors in Figure 5(4), intermediate compounds having unrealistic structures are often included in candidate pathways due to the ambiguous characteristics of the latent space of the JT-VAE. To eliminate such a pathway, we calculate changes in the molecular weight from a substrate and product at each segment of the candidate pathways. Namely, we omit a pathway including a segment having greater change in molecular weight than the predefined threshold.

By repeating steps (i)−(v) several times, it is possible to obtain candidate pathways from the start compound to target compound.

#### 2.2.4 Pathway scoring of candidate pathways

By using the reaction-feature vectors, we also developed a pathway-scoring method to evaluate the feasibility of candidate pathways designed according to the method explained in the previous section. Reaction-possibility prediction is carried out using the voting scheme that averages the outputs of the sets of discriminators trained with different datasets.

The voting scheme is an effective method for outputting the prediction values in terms of reducing the rejection rate and/or improving the accuracy rate (Battiti and Colla, 1994). A general binary classification cannot deal well with real reactions that are mistakenly judged as virtual or reactions that have the possibility of reaction that would actually occur but tagged as a virtual reaction. ‘Virtual’ means that the reaction is virtually calculated on computer and not registered in KEGG. To solve this problem, we avoid complete rejection by using the voting scheme for an output of an ensemble of NNs. That is, an output is not as a value of real (1) or virtual (0) value but a reaction-possibility value from 0.0 to 1.0 with ambiguity.

**Fig. 6.**
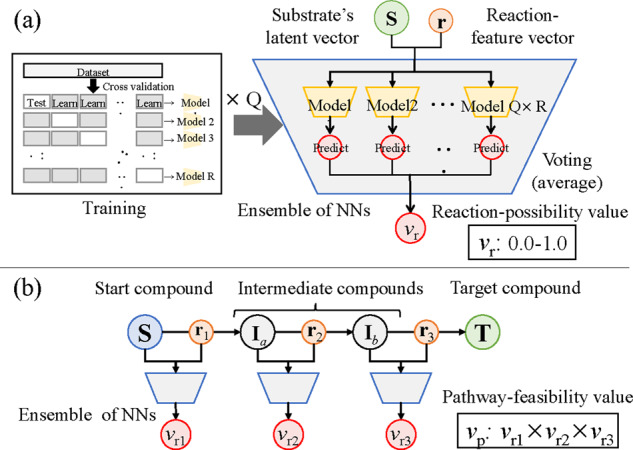
Pathway-scoring method of candidate pathways. (**a**) Ensemble of neural networks (NNs) is used for predicting reaction-possibility value. Multiple NN model weights are obtained from training using each dataset. Each NN outputs 0 or 1. Reaction-possibility value *v_r_* from 0.0 to 1.0 is finally obtained using voting scheme. (**b**) This is example of pathway-feasibility value *v_p_* by multiplying three reaction-possibility values of reaction feature vectors ($v_{r1},\;v_{r2}$ and $v_{r3}$)

Figure 6a illustrates the ensemble of NNs and the training of each NN. Each reaction-possibility value *v_r_* is acquired from the ensemble of NNs. Each NN takes the input as a pair of a reaction-feature vector and substrate-latent vector and outputs 0 or 1. In training, multiple weights are acquired when performing *R*-fold cross validation for each dataset (the number of datasets is *Q*). The registered enzymatic-reaction data are set as real data (labeled as 1.0) and non-registered data are set as virtual data (labeled as 0.0). The total number of NN models is *Q *×* R*. The average of these outputs is taken, then a *v_r_* is calculated from 0.0 to 1.0. 

Figure 6b shows how the pathway-feasibility value *v_p_* of each candidate pathway is obtained when three reactions are selected in the pathway from the start compound to the target compound. When three reactions are selected, there will be two intermediate compounds. That is, in the latent space, the pathway-feature vector from the latent vector **S** of start compound to that of target compound **T** are represented by three reaction feature vectors ($r_{1},\;r_{2}$ and $r_{3}$). In addition, two intermediate compounds are represented as $I_{a}$ and $I_{b}$. In each reaction, the latent vector of the substrate and the reaction feature vector are input to the above ensemble NN to obtain the *v_r_*. By multiplying all the obtained *v_r_*s, the *v_p_* is obtained.

The candidate pathways are then sorted by the score *s* calculated with the absolute error |e| and *v_p_*, as shown in Equation (6).

(6) $$s=\frac{\left|e\right|}{v_{p}}$$

## 3 Results

### 3.1 Datasets and VAE training

The VAE-training dataset of SMILES consisted of the ZINC dataset (Sterling and Irwin, 2015) used in the JT-VAE and compound data acquired from metabolic-pathway DB, KEGG. The SMILES strings of the compounds of the metabolic-pathway DBs were acquired from PubChem (Kim *et al.*, 2016) and ChEBI (Degtyarenko *et al.*, 2007). In the training dataset, compounds containing ‘*’ indicating a wild card and ‘.’ of an ionic bond were excluded. Over 260K pieces of compound data were prepared under these conditions. The number of training epochs was set as 10. By applying tree decomposition over 260K molecules, we collected our vocabulary set *V* of size |V|=1279. The hidden state dimension was set as 450 for all modules in JT-VAE and the latent bottleneck dimension was set as 56 by referring to JT-VAE (Jin *et al.*, 2018).

The enzymatic-reaction dataset for pathway design consisted of 9794 pieces of reaction data acquired from the metabolic-pathway DBs. Each piece of data includes an EC number and reaction pair of the main substrate and main product. By using the trained encoder of the JT-VAE, the latent vectors of compounds were acquired from the metabolic-pathway DBs (Kanehisa and Goto, 2000) and compound DBs (Degtyarenko *et al.*, 2007; Kim *et al.*, 2016). The reaction vectors of the enzymatic-reaction dataset were generated using the chemical-latent vectors. Then, each reaction vector was recorded to the dataset and assigned an EC number.

In the training of the NN-based reaction-possibility prediction for pathway scoring, four types of datasets were used. The details of virtual datasets are given in Section 3.5.1.

### 3.2 Reaction representation

#### 3.2.1 Reconstruction performance of metabolic pathway compounds

Although a study on the JT-VAE using the ZINC dataset reported that the reconstruction accuracy was ∼70% (Jin *et al.*, 2018), the reconstruction accuracy of the KEGG compound dataset we used was ∼56%. KEGG compounds contain relatively large numbers of macrocyclic and long-chain compounds. The reconstruction of these compounds has bad chemistry with the JT-VAE. This is because the estimation becomes difficult when the number of neighbors in the junction tree increases or the number of prediction steps increases.

#### 3.2.2 Enzymatic reaction classification performance

**Fig. 7.**
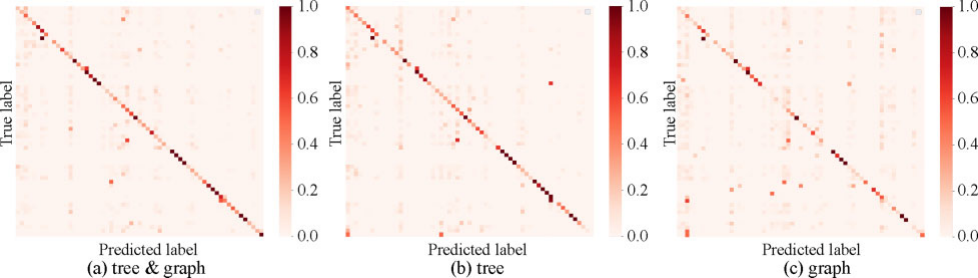
Confusion matrices for classification accuracies of each EC number class (digit: 2, classifier: LDA). (**a**) Tree & graph means that combination of tree- and graph-latent vectors of JT-VAE were used, (**b**) tree-latent vector was used and (**c**) graph-latent vector was used

EC number classes were set as the same EC number class of each digit (i.e. one digit: ECX; two digits: ECX.X; three digits: ECX.X.X). Each EC number (one digit) had the following number of reaction-data pieces (Table 1). The reaction-feature vectors of the same EC number class should be distributed closely in the feature space because the same type of enzyme may work for the same type of structural change. To examine reaction-feature representations useful for pathway design, a combination of tree- and graph-latent vectors (normal), tree-latent vector and graph-latent vector of the JT-VAE were compared on the basis of the classification accuracies of reaction-feature vectors by using linear discriminant analysis (LDA). Figure 7 shows the classification results from the LDA for the reaction-feature vectors of among latent vectors. The confusion matrices of two digits for each vector were calculated by aggregating the confusion matrices of the results under the condition that the digit of the EC number was three and the number of data pieces was more than one. As a result, the classification accuracies of the tree-latent vector were equal to the accuracies of combined tree- and graph-latent vectors. It is also suggested that classifying the reaction-feature vector using only the graph-latent vector was much more difficult than using the tree-latent vectors. These results indicate that the use of tree-, or both tree and graph-latent vectors can determine the characteristics of each enzyme class. From the above results, tree-latent vectors were used for the pathway design of candidate pathways. The calculation cost of pathway design can be reduced as compared to using tree- and graph-latent vectors. 

**Table 1.** Number of data pieces for each EC number class (one digit)

EC number class	1	2	3	4	5	6	7
Number of data pieces	3845	2667	1287	1158	488	344	4

### 3.3 Reconstruction results after enzymatic reaction in latent space

**Fig. 8.**
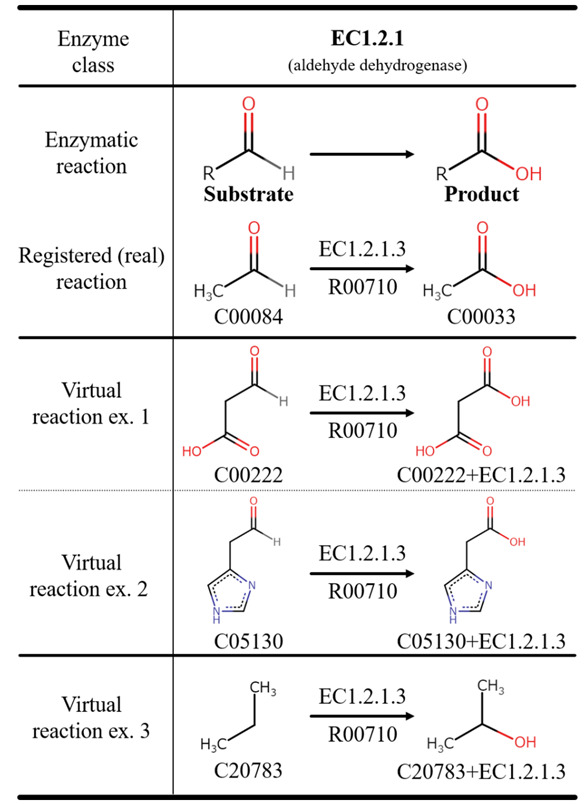
Enzymatic reactions EC1.2.1.3 in the latent space

By using the JT-VAE to decode the latent vectors of products obtained by adding reaction-feature vectors to the latent vectors of substrates, the structures of the products in which the desired structural change occurred in the same enzyme class were obtained. Figure 8 shows example results of the enzymatic reactions EC1.2.1 called dehydrogenase in the latent space. ‘Registered reaction (real)’ means that the reaction is registered in the KEGG. ‘Virtual’ means that the reaction is virtually calculated on computer. The ‘Registered reaction (real)’ of EC1.2.1.3 registered in KEGG is a reaction from which carboxylate (KEGG Compound ID: C00033) is produced from aldehyde (KEGG Compound ID: C00084). Figure 8 shows three examples of enzyme reactions in latent space by using the reaction-feature vector of EC1.2.1.3. The results in ex. 1 and ex. 2 in the figure show that the enzymatic reactions have the same structural changes as ‘Registered reaction (real)’. In addition, the result in ex. 3 shows that the enzymatic reaction cannot occur biologically because the substrates do not have essential structures for catalyzation of the enzymes. 

### 3.4 Pathway design of candidate pathways

We designed candidate pathways under the following conditions. Only tree-latent vectors were used for the selection of the reaction-feature vectors. The DE’s parameters, i.e. scaling parameter *F* and crossover rate *CR*, were set as 0.5 and 0.5, respectively. The constant parameter *C* of Equation (5) was set to 1000.0. The error threshold Th was set as 50.0. The number of populations was set to 2000, and that of generations was set to a maximum of 50. The threshold of molecular weight check for omitting unrealistic pathways was set as the amount of molecular weight change ±3 between the main substrate and the main product of the registered reaction corresponding to the selected EC number.

**Fig. 9.**
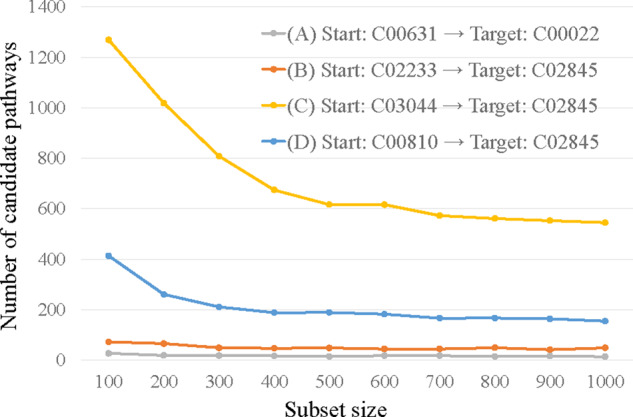
Transition in number of candidate pathways when number of repetitions was set to 2000 and subset size was changed from 100 to 1000 in steps of 100. The transitions are (A) pathway from C00631 to C00022 including two registered reactions from (B) pathway from C02233 to C02845 including one non-registered reaction, (C) pathway from C03044 to C02845 including one non-registered reaction and (D) pathway from C00810 to C02845 including one registered reaction and non-registered reaction

We confirmed the change in the number of candidate pathways with respect to the subset size. Figure 9 shows the transitions in the number of candidate pathways when the number of repetitions was set to 2000 and the subset size was changed from 100 to 1000 in steps of 100. Each pathway included one or two reaction steps to the target compound. The transitions are (A) two registered reactions, (B), (C) one non-registered reaction (two types) and (D) a registered reaction and non-registered reaction. The number of explored candidate pathways tended to decrease as the size of the subset increased. This is because the larger the subset size, the higher the probability that the subset will contain a particular desired reaction combination. In the examples of the pathways including one or two reaction steps, the reduction rate tended to slow at subset sizes around from 400 to 500. The number of candidate pathways varied widely from pathway to pathway. The number of candidate pathways tended to increase as the number of combination patterns of enzyme reactions meeting a predetermined threshold and having similar features increased. The pathways of (A) and (B) had a smaller number of candidate pathways than those of (C) and (D). Moreover, the difference between pathway of (C) and pathway of (D) was stable when the subset size was over 500. The number of candidate pathways decreased as the number of reactions increased. Hence, it is necessary to set short pathways to explore many candidate pathways with this subset method. The difference in the number of candidate pathways is related to the number of structures that exhibit the same structural change. 

### 3.5 Pathway scoring of candidate pathways

#### 3.5.1 Results of reaction-possibility prediction

We applied the reaction-possibility prediction method using the ensemble of NNs to the enzymatic reactions that include both registered (real) reactions and virtual reactions. As described in Section 3.5.1, a reaction-feature vector and the tree- and graph-latent vector of a substrate were used for input. We considered the following two terms regarding the input.

1. Whether an enzyme reaction pair that constituted the reaction feature vector is real or virtual.
2. Whether the substrate constituted the reaction-feature vector.

Therefore, we fist prepared the following four types of datasets for training the NNs.

- ‘Real’ dataset consisting of a real enzymatic reaction-feature vector registered in KEGG and the latent vector of the substrate used for calculating the reaction-feature vector of the enzymatic reaction (Real pair, Substrate in).
- ‘Virtual-1’ dataset consisting of a real enzymatic reaction-feature vector obtained from KEGG and the latent vectors of substrates not used for calculating the reaction-feature vector of the enzymatic reaction (Real pair, Substrate out).
- ‘Virtual-2’ dataset consisting of a virtual enzymatic reaction-feature vector consisting of the latent vector of the substrate and product, which were randomly selected, and the latent vector of substrate used for calculating the reaction-feature vector of the enzymatic reactions (Virtual pair, Substrate in).
- ‘Virtual-3’ dataset consisting of a virtual enzymatic reaction-feature vector consisting of the latent vector of the substrate and product, which were randomly selected, and the latent vector of the substrate which was not used for calculating the reaction-feature vector (Virtual pair, Substrate out).

The ‘Real’ dataset had 9794 pieces of enzymatic reaction data registered in KEGG. In addition, the number of each virtual type of dataset was 10. Each virtual dataset had 10,000 pieces of data. Therefore, one training dataset combining real and virtual data consisted of 19,794 pieces of data. In the training, 150 weights were acquired when carrying out 5-fold-cross validation for each dataset.

Each NN had three full-connected middle layers (64, 32, 8). All activation functions were set as the Rectified Linear Unit. Each model outputs 0 or 1 for each input. The average of these 150 outputs was taken, and finally a reaction-possibility value was calculated from 0.0 to 1.0.

Table 2 lists the results of the average and standard deviation of the reaction-possibility prediction for each type of data. We confirmed that the scores of the real and virtual data significantly differed. The average value of the ‘Real’ data was close to 1.0. However, those of the ‘Virtual-1’ and ‘Virtual-2’ data were close to or less than 0.5. The values of ‘Virtual-3’ data were very small. The more realistic elements were included, the higher the possibilities of reactions were, and the virtual data were not completely 0.0. This is a reasonable result because a reaction that may be determined to be real is actually included when estimating an unregistered reaction. 

**Table 2.** Results of reaction-possibility prediction (max: 1.0; min: 0.0)

	Substrate in	Substrate out
Real pair	0.99±0.02	0.54±0.23
Virtual pair	0.35±0.17	0.09±0.12

#### 3.5.2 Results of candidate-pathway scoring

**Fig. 10.**
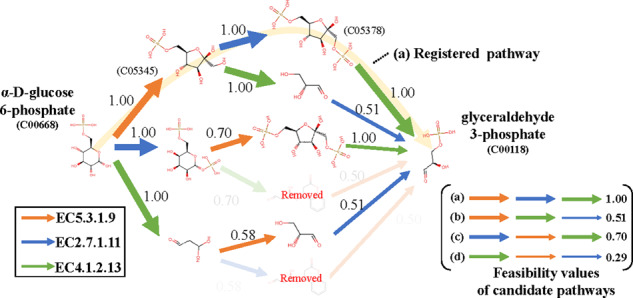
Results of candidate-pathway scoring. Pathway from *α*-d-glucose 6-phosphate (KEGG Compound ID: C00668) to glyceraldehyde 3-phosphate (KEGG Compound ID: C00118) was used. Selected enzymatic reactions were EC5.3.1.9, EC2.7.1.1 and EC4.1.2.13. There were six combinations. Each line indicating each reaction-feature vector is in different color, and thickness of line corresponds to value of each reaction possibility

The performance of reaction-possibility prediction was verified using a part of the ‘glycolysis’ pathway. Specifically, the feasibility value of each candidate pathway acquired using the reaction-possibility prediction method was verified when the pathways were designed based on the condition that all reaction feature vectors are used in the pathway from *α*-d-glucose 6-phosphate (KEGG Compound ID: C00668) to glyceraldehyde 3-phosphate (KEGG Compound ID: C00118). That is, the selected enzymatic reactions were EC5.3.1.9, EC2.7.1.1 and EC4.1.2.13. Figure 10 shows the results of the pathway-feasibility values of candidate pathways. It should be noted that, after reconstruction of compounds, some pathways may be removed by pruning the pathway based on the amount of change in molecular weights. We confirmed that the registered pathway had the highest feasibility value and that pathway scoring indicates that pathway pruning using the reaction-possibility value can also be applied. 

### 3.6 Feasible pathway exploration

**Fig. 11.**
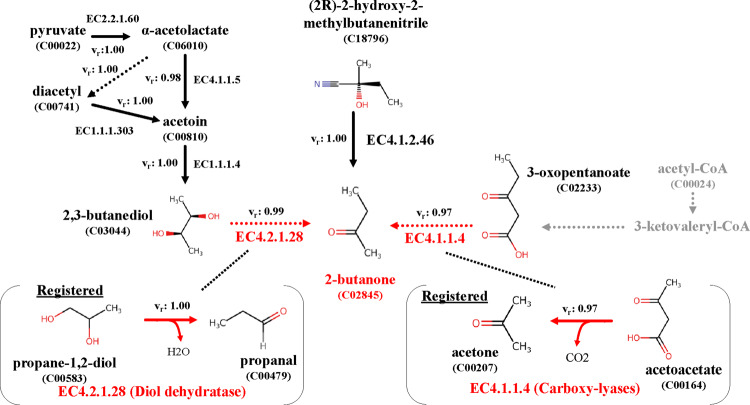
Results of exploring feasible pathways. Pathway from Pyruvate (KEGG Compound ID: C00022) to 2-butanone (KEGG Compound ID: C02845) and pathway from acetyl-CoA (KEGG Compound ID: C00024) to 2-butanone are reported in Srirangan *et al.* (2016) and Chen *et al.* (2015), respectively, but both reactions from precursors to 2-butanone are not registered in KEGG. Non-registered reactions are represented as red dotted lines. Moreover, *v_r_*s are reaction-possibility values. Both reactions were explored using proposed technique

We applied the proposed technique to non-registered pathways to verify its performance. Figure 11 shows two enzymatic reactions of validation pathways that are non-registered pathways for producing the target compound 2-butanone (KEGG Compound ID: C02845) reported in previous studies (Chen *et al.*, 2015; Srirangan *et al.*, 2016). 

We first investigated the reaction-feature vectors closest to the two types of reported non-registered reactions in each enzymatic reaction class of three digits. We then verified whether the pathways could be explored using the proposed technique. Each reaction-feature vector was calculated from the difference between the latent vector of each target compound and those of the precursor. The pathway design of candidate pathways was carried out using only the tree-latent vector of the JT-VAE. The pathway score and each reaction-possibility value of the enzymatic reactions were also output with the reaction-possibility prediction method.

We explored feasible pathways connecting metabolic pathways, as shown in Figure 11. Validation pathway A was reported by Srirangan *et al.* (2016). This pathway includes the non-registered enzymatic reaction in which 2-butanone is generated from 3-oxopentanoate (KEGG Compound ID: C02233). The EC number of the corresponding reaction is 4.1.1.4. The enzymatic reaction of EC4.1.1.4 registered in KEGG is a reaction from which acetone (KEGG Compound ID: C00207) is produced from acetoacetate (KEGG Compound ID: C00164). This pathway reports a pathway involving a compound with CoA, but since it is difficult to target a long compound such as CoA with the JT-VAE, we explored from the precursor, 3-oxopentanoate. Namely, we applied the proposed technique to the pathway when 2-butanone was set as the target compound and 3-oxopentanoate as the precursor was set as the start compound. Validation pathway B was reported by Chen *et al.* (2015). This pathway includes the non-registered enzymatic reaction from which 2-butanone is produced from 2,3-butanediol (KEGG Compound ID: C003044). The EC number of the corresponding reaction is 4.2.1.28. The enzymatic reaction of EC4.2.1.28 registered in KEGG is a reaction from which propanal (KEGG Compound ID: C00479) is produced from propane-1,2-diol (KEGG Compound ID: C00583). For validating pathway B, pathway exploration was conducted with the proposed technique using acetoin (KEGG Compound ID: C00810), which is a precursor of the precursor, as a start compound. We confirmed that each reaction-feature vector generated by the substrate and product described in each study was very similar to the reaction feature-vector of the EC number described in those studies. In both enzymatic reactions, the EC number of the most similar reaction-feature vector in the relevant EC number class (three digits) matched the number described in those papers. Moreover, by using the proposed technique, the feasible pathways including potential pathways reported in the previous research (Chen *et al.*, 2015; Srirangan *et al.*, 2016) could be explored, as shown in Figure 11, i.e. red dotted lines. Pathway B from pyruvate to 2-butanone includes four or five reactions. However, when 2-butanone was explored as the target compound, the correct pathway was obtained for one or two step reactions to 2-butanone. Namely, we obtained correct pathways when 2,3-butanediol or acetoin was set as the start compound. We confirmed that if the number of reactions is three or more, the probability that the correct reactions were included in the subset decreases; thus, exploration becomes difficult.

## 4 Discussion and conclusions

We proposed a feasible-pathway-exploration technique, which involves (i) reaction representation using chemical latent space for an enzymatic reaction on a computer system, (ii) candidate-pathway design using a DE algorithm by combining potential enzymatic reactions and (iii) pathway scoring using an NN-based reaction-possibility prediction method for determining the pathway-feasibility values of the candidate pathways. We applied the proposed technique to the non-registered pathways relevant to the production of 2-butanone. The proposed technique explored feasible pathways including non-registered enzymatic reactions.

From the results shown in Figures 8 and 11, the same structural change as the relevant enzyme reaction can occur by adding the reaction-feature vector to the latent vector of the substrate. As shown in Figure 8 for ‘Virtual 3’, deviating reactions from the enzyme-reaction rules were confirmed because the enzyme-reaction rules were not applied, although the degree of freedom of the reaction representation was high. We removed the pathways including such reactions based on the amount of change in molecular weight. With a hybrid method applying the minimum enzyme-reaction rules to reaction representation, a more accurate solution can be expected to this problem.

The tree-latent vector of the JT-VAE used for pathway exploration was useful for classification of enzyme reactions and pathway design, confirming that it can capture the substrate specificity of enzyme reactions. This is because the feature-tree technique (Rarey and Dixon, 1998), which deals with substructures as chunks, can capture the similarity of changes in the overall backbone structure. Moreover, in the pathway design of candidate pathways, the binary DE algorithm was simply applied to the NLP problem whose dimension was large in combination with the subset method. This is a very effective method in the exploration of pathways including one or two reactions. Namely, the feasible pathways could be explored when the precursor of a target compound or the compound before the precursor was set as the start compound. The use of the subset method raised a problem in that an effective solution could not be provided unless the corresponding reaction was included due to the increase in the number of reactions. To solve this problem, clustering the features of the reaction in advance and applying a multi-step search using the center vector are effective. This enables searches that target all reactions while maintaining search efficiency. A method with which the reaction-feature vector DB is formed into a tree structure is also effective.

From the results in Figure 11, the feasibility values of candidate pathways using the NN-based reaction-possibility prediction method were near 1.0 for actual pathways and reactions not registered in KEGG but reported in the paper. The values were lower for non-registered reactions not reported. Therefore, we succeeded in making the reaction-possibility prediction method based on the registered-reaction DB. In pathway scoring, it is necessary to score with higher accuracy by gathering the enzymatic reactions in other DBs and papers. It is effective not only to judge whether there is a registered enzymatic reaction but also to carry out training with an index such as a physical quantity relating to the enzymatic reaction. For example, it may be possible to incorporate indicators such as toxicity and naturalness.

Regarding future challenges for chemical VAEs, a technique for improving compound-reconstruction accuracy and dealing with the compounds excluded in this paper is necessary. There is a need for technology that can use long-chain compounds, which have long SMILES character strings, compounds containing macrocycles and those represented by ionic bonds that cannot be ignored in metabolic pathways. As a state-of-the-art technique, a hyper-graph grammar for chemical structures was proposed (Kajino, 2019). This technique has higher compound-reconstruction accuracy than using JT-VAE. We will improve the proposed technique so that multi-step pathways can be explored more accurately.

## Data Availability

The datasets used in this study are available from the corresponding author, T. Fuji (taiki.fuji.mn@hitachi.com), upon reasonable request.
